# Sex differences in frailty of geriatric outpatients with type 2 diabetes mellitus: a multicentre cross-sectional study

**DOI:** 10.1038/s41598-022-20678-7

**Published:** 2022-09-27

**Authors:** Huan Thanh Nguyen, An Huu Nguyen, Phuong Thi My Le

**Affiliations:** Department of Geriatrics and Gerontology, University of Medicine and Pharmacy at Ho Chi Minh City, 217 Hong Bang Street, Ward 11, District 5, Ho Chi Minh City, Vietnam

**Keywords:** Type 2 diabetes, Geriatrics

## Abstract

Frailty and type 2 diabetes mellitus (T2DM) can occur concurrently and are increasingly prevalent in older populations. There is a marked variability in frailty progression between men and women. This study aimed to investigate sex differences in the prevalence and factors associated with frailty in older outpatients with T2DM. This multicentre cross-sectional study included 638 outpatients (aged ≥ 60 years; median age 71 years [interquartile range, 66–77]; male, 55.5%) and was conducted from January 2019 to July 2020. Frailty was assessed using the Fried frailty phenotype. Factors associated with frailty were assessed using a logistic regression analysis. The overall frailty prevalence was 28.2% (men, 26.8%; women, 29.9%; *P* = 0.388). In the adjusted model, the factors associated with greater odds of being frail were older age (odds ratio [OR], 1.08; 95% confidence interval [CI], 1.05–1.11; *P* < 0.001) and body mass index (BMI) less than 20 kg/m^2^ (OR, 1.96; 95% CI, 1.16–3.32; *P* = 0.012). Higher education (OR, 0.64; 95% CI, 0.42–0.98; *P* = 0.041) and productive work (OR, 0.11; 95% CI, 0.03–0.36; *P* < 0.001) were protective factors against frailty. Frailty was associated with all four factors in women, but only with older age and productive work in men. Our study found that the prevalence of frailty in older outpatients with T2DM was 28.2%, though not significantly different between men and women. While older age and BMI less than 20 kg/m^2^ can increase the odds of frailty, and higher education and productive work can decrease the odds of frailty in women, only age and productive work were associated with frailty in men with T2DM.

## Introduction

Frailty is an important geriatric syndrome characterised by increased vulnerability and decreased ability of older adults to regain homeostasis following stressor events^1^. The prevalence of frailty increases with age, and the presence and severity of this clinical state can influence the manifestation and clinical outcomes of multiple comorbidities, including endocrine and cardiovascular diseases^2,3^. Frailty is also a risk factor for the development of major adverse cardiovascular events^4^. Fortunately, the progression of frailty can be delayed by early diagnosis and appropriate intervention^5^.

Type 2 diabetes mellitus (T2DM) is a major global health and economic burden due to an ageing population^6^. Management of T2DM in older adults is difficult owing to the coexistence of geriatric syndromes, such as polypharmacy, multimorbidity, falls, delirium, and frailty^7^. Frailty is not only an independent predictor of incident T2DM in older adults^8^, but is also associated with mortality, complications, and lower quality of life in people with diabetes^2^. Although T2DM was confirmed not to be significantly correlate with frailty in the geriatric patients^9^, the identification and assessment of frailty is increasingly recognised in recent clinical guidelines for diabetes to determine targets and therapeutic approaches for older patients with T2DM^10,11^.

Accumulating evidence has shown that there is a male–female health-survival paradox in the older population. Although women have a longer life expectancy than men^12^, systematic reviews of community-dwelling populations have found greater levels of disability, more comorbidities, and a higher prevalence of frailty in older women than in older men^13,14^. The discrepancy between health and survival may suggest that women tolerate frailty better than men. It may also be related to the differences in social, behavioural, and biological factors between the two groups^15,16^.

Vietnam, a lower middle-income country, entered a growth phase in their ageing population in 2011. In 2019, people aged ≥ 60 years accounted for 13.2% (men, 6.1%; women, 7.2%) of the total Vietnamese population^17^. The country has also undergone an epidemiological transition, with health alterations from infectious diseases to non-communicable diseases^18^. In Vietnam, 6% of the total population had diabetes^19^, and among older individuals, the diabetes rate was approximately 29%^20^. However, little clinical information is available to understand whether the characteristics of frailty in older adults with T2DM differ between the sexes. Therefore, the aim of this study was to investigate sex differences in the prevalence and factors associated with frailty in older outpatients with T2DM.

## Material and methods

### Study design and participants

This cross-sectional study was conducted in outpatients aged ≥ 60 years with T2DM at three geriatric clinics from January 2019 to July 2020. Participants met the inclusion criteria if they were diagnosed with T2DM for one year or more before enrolment based on a fasting plasma glucose level of ≥ 7.0 mmol/L after no caloric intake for at least 8 h and/or haemoglobin A1c (HbA1c) level of ≥ 6.5%. To ensure consistent management, trained geriatricians treated all patients for T2DM with any medication and with individualised HbA1c targets based on the recent guidelines of the European Society of Cardiology and European Association for the Study of Diabetes^11^. Exclusion criteria were hospital admission, active malignancy, serious mental condition, or heart failure categorised as New York Heart Association class III-IV. All participants provided written informed consent and underwent a comprehensive geriatric assessment, including demographic characteristics, Fried frailty phenotype, and comorbidities. Our study follows The Strengthening the Reporting of Observational Studies in Epidemiology (STROBE) statement^21^.

### Sample size calculation

The sample size was calculated for the first aim of this study using a single population proportion formula: n = Z^2^_1−α/2_*[p*(1 − *p*)/d^2^], where n = the required sample size, Z_1−α/2_ = 1.96 (with α = 0.05, and 95% confidence interval), *p* = prevalence of frailty in older outpatients with T2DM in Vietnam, and d = precision (assumed as 0.04). Because the prevalence is unknown, we set *p* as 0.5 to obtain the maximum possible value of p*(1 − *p*) as 0.25. This study required a minimum of 600 participants.

### Variables and definitions

Geriatricians managing the patients were responsible for collecting demographic data, clinical characteristics, and measuring body weight and height. Self-reported information was obtained on age, sex, marital status, level of education, living status (alone or with anyone), and productive work. The patients’ educational level was classified as lower education (no school, elementary, and junior high school) or higher education (senior high school, university, and above). Productive work was defined as participants having any form of paid or unpaid job. Comorbidities were obtained from interviews and electronic medical records. Polypharmacy and multimorbidity data were collected based on the prescriptions of the patients. Polypharmacy was defined as five or more medications^22^. Multimorbidity was defined as the presence of two or more chronic diseases^23^.

Body mass index (BMI) was calculated as the quotient of body weight (kg) and height (m^2^). Body weight and height were measured following a standardised protocol using identical equipment at all study sites. Because of the differences in body mass index classifications between the World Health Organization and Asia–Pacific guidelines, the BMI of our patients was categorised into five groups (< 20, 20–24.9, 25–29.9, 30–34.9, and ≥ 35 kg/m^2^) according to a previous study investigating the relationship between frailty and BMI in older people^24^.

### Assessment of frailty

Patients were physically examined and placed into one of three categories using the Fried frailty phenotype: frail (≥ 3 criteria present), pre-frail (1–2 criteria present), or non-frail (0 criteria present)^25^. A Vietnamese version of Fried criteria was carefully explained to each patient, and caregivers were asked to ensure that the reporting context was correct (Table S1). The five components are as follows.

1. Unintentional weight loss of ≥ 4.5 kg or ≥ 5% body weight in the past year.
2. Weakness: Grip strength of the dominant hand was measured once in the sitting position using a Jamar 5030-J1 hydraulic hand dynamometer (JLW Instruments, Chicago, IL 60,607, United States) with relaxed shoulders and encouragement. Weakness was defined as the lowest quintile of grip strength, stratified according to sex and body mass index (BMI). The BMI cut-off points were ≤ 29.0, ≤ 30.0, and ≤ 32.0 kg for BMI ≤ 24.0, 24.1–28.0, and > 28.0, respectively, in men and ≤ 17.0, ≤ 17.3, ≤ 18.0, and ≤ 21.0 kg for BMI ≤ 23.0; 23.1–26.0; ≤ 26.1–29.0, and > 29.0, respectively, in women.
3. Exhaustion: Two questions from the Centre for Epidemiologic Studies Depression Scale were used: ‘I felt that everything I did was an effort last week’ and ‘I could not get going last week’^25^. Participants answering ‘frequently’ or ‘always’ to at least one of these two questions were categorised as having met the criterion for exhaustion.
4. Slowness: The walking time of participants over a 4.57 m distance was adjusted for gender and height. The cut-off points for slow walking speed were established as height ≤ 173 cm and time ≥ 7 s (equivalent to 0.65 m/s) or height > 1.73 cm and time ≥ 6 s (equivalent to 0.76 m/s) for men, and height ≤ 1.59 cm and time ≥ 7 s (0.65 m/s) or height > 1.59 cm and time ≥ 6 s (0.76 m/s) for women.
5. Low physical activity: We used the short version of the Minnesota Leisure Time Activity questionnaire, which included questions on 18 activities: walking, chores, mowing the lawn, raking, gardening, hiking, jogging, biking, exercise cycling, dancing, aerobics, bowling, golf, singles tennis, doubles tennis, racquetball, callisthenics, and swimming^25^. The total weekly kilocalories of physical activity expenditure were calculated using a standardised algorithm. Low activity levels were defined as < 383 kcal in men and < 270 kcal in women.

### Statistical analysis

All collected data were analysed using the IBM SPSS Statistics for Windows, version 25 (IBM Corp., Armonk, NY, USA). Categorical variables were described as frequencies and percentages (%). The Kolmogorov–Smirnov test was conducted to assess the distribution of continuous variables. Continuous variables were described using means and standard deviations for normal distribution and median and interquartile range (IQR) (25–75th percentile) for non-normal distribution. Chi-square test or Fisher’s exact test was used to compare categorical variables. Student’s t-test or one-way ANOVA was used to determine the statistical significance of the difference between two or more study group means. The Mann–Whitney and Kruskal–Wallis tests were used to compare two or more groups with non-normal distribution. To determine factors associated with frailty, the non-frail and pre-frail groups were pooled together in a non-frail group. Univariate logistic regression analysis was performed to identify potential factors associated with frailty. Variables with *P* values < 0.2 in the univariate analysis, were selected for multivariate logistic regression. All variables were examined for their interaction and multicollinearity. Multicollinearity was assessed using variance inflation factor (VIF)^26^. Multicollinearity is present when the VIF is higher than 5. All tests were two-sided, and the significance level was set at *P* < 0.05.

### Ethical approval

The present study was conducted in accordance with the guidelines of the Declaration of Helsinki and approved by the Ethics Committee of the University of Medicine and Pharmacy at Ho Chi Minh City, Vietnam (approval number, 01/QĐ-ĐHYD [approved on January 16, 2019]).

### Informed consent

Informed consent was obtained from all individual participants included in the study.

## Results

### Prevalence of frailty in older outpatients with T2DM

**Figure 1:**
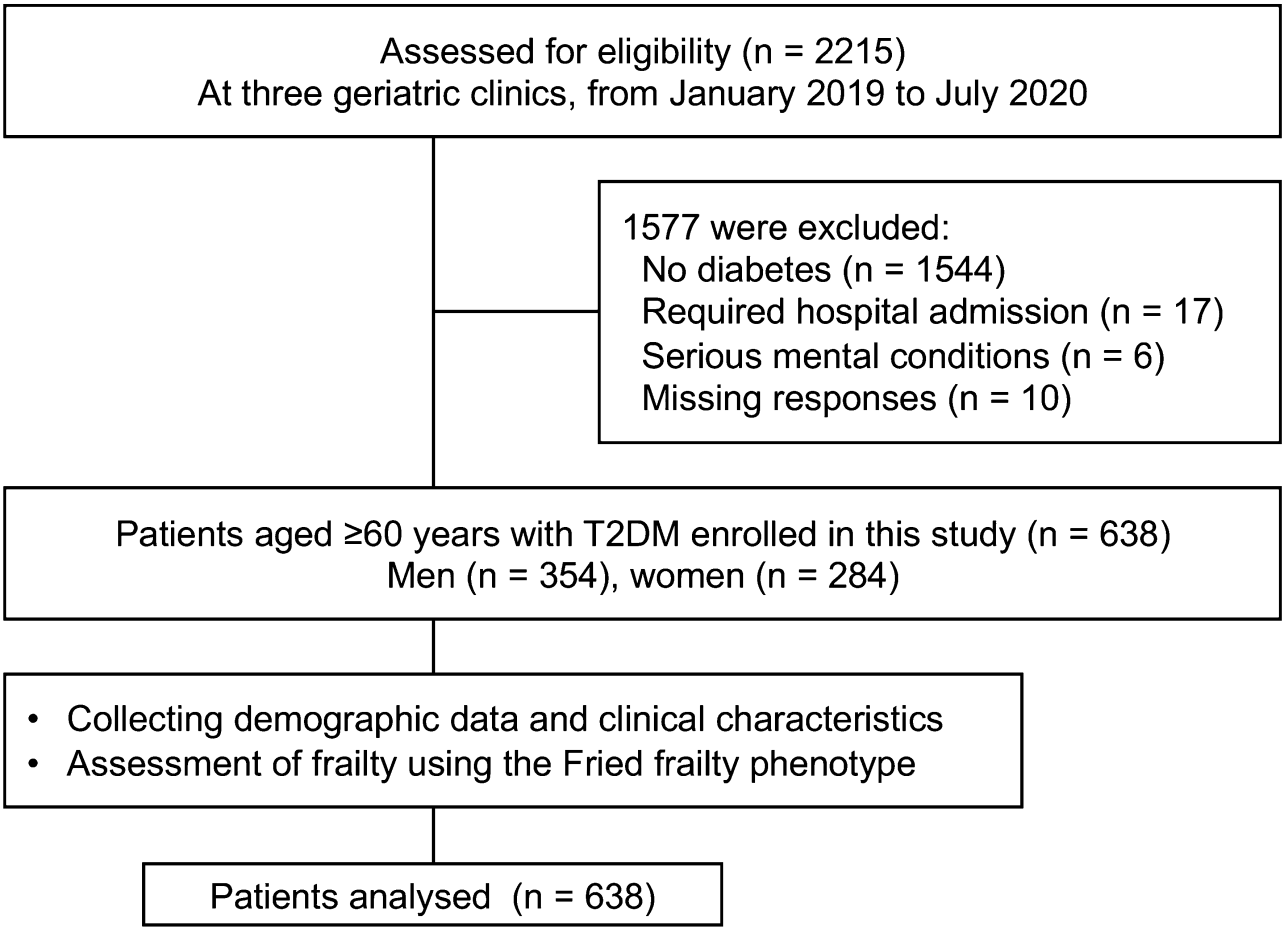
Flow chart of the enrollment of participants.

Of the 2215 patients admitted to our geriatric clinics during the study period, 1577 were excluded because they did not have diabetes (1544 patients), required hospital admission (17 patients), had serious mental conditions (6 patients), or had missing responses (10 patients). Figure 1 shows the flow diagram for the sample selection. The 638 older patients with T2DM enrolled in this study had a median age of 71 years (IQR: 66–77; range, 60–92) and male predominance (55.5%). The overall prevalence of non-frail, pre-frail, and frail categories was 17.9% (n = 114), 53.9% (n = 344), and 28.2% (n = 180), respectively.

### Differences in main characteristics between men and women

Table 1 summarises the characteristics of the patients according to sex. The men and women did not differ in terms of living status, productive work, polypharmacy, multimorbidity, and some medical disorders (i.e. hypertension, heart failure, stroke, and chronic pulmonary diseases). However, there were significant differences in age, marital status, level of education, BMI group, coronary artery disease, osteoarthritis, and chronic kidney disease between the two groups. The women’s group was significantly older than the men’s group, and there were more women than men aged 75 years and over (46.1% vs. 31.1%, *P* < 0.001). Importantly, the women’s group had a significantly lower rate of higher education than the men’s group (37.7% vs. 76.3%, *P* < 0.001). The two most common conditions in both groups were hypertension and osteoarthritis, followed by chronic kidney disease in the men’s group and coronary heart disease in the women’s group.

**Table 1:** Characteristics of the participants according to sex.

Characteristics	All (n = 638)	Men (n = 354)	Women (n = 284)	*P*-value
Age, years	71.0 (66–77)	70.5 (65–76)	72.5 (67–78)	0.002
Age ≥ 75 years, n (%)	241 (37.8)	110 (31.1)	131 (46.1)	< 0.001
**Marital status, n (%)**				< 0.001
Single	7 (1.1)	0 (0)	7 (2.5)	
Married	477 (74.8)	328 (92.7)	149 (52.5)	
Divorced/Widowed	154 (24.1)	26 (7.3)	128 (45.0)	
Higher education, n (%)	377 (59.1)	270 (76.3)	107 (37.7)	< 0.001
Living alone, n (%)	72 (11.3)	34 (9.6)	38 (13.4)	0.170
Productive work, n (%)	74 (11.6)	43 (12.1)	31 (10.9)	0.720
BMI, kg/m^2^	22.9 ± 3.1	23.0 ± 2.9	22.8 ± 3.4	0.369
**BMI groups, n (%)**				0.001
< 20	101 (15.8)	51 (14.4)	50 (17.6)	
20–24.9	379 (59.4)	207 (58.5)	172 (60.6)	
25–29.9	147 (23.1)	95 (26.8)	52 (18.3)	
30–34.9	11 (1.7)	1 (0.3)	10 (3.5)	
Polypharmacy, n (%)	367 (57.5)	212 (59.9)	155 (54.6)	0.205
Multimorbidity, n (%)	591 (92.6)	327 (92.4)	264 (93.0)	0.898
**Medical disorders, n (%)**				
Hypertension	566 (88.7)	320 (90.4)	246 (86.6)	0.170
Coronary artery disease	172 (27.0)	65 (18.4)	107 (37.7)	< 0.001
Heart failure	8 (1.3)	4 (1.1)	4 (1.4)	0.753
Stroke	21 (3.3)	14 (4.0)	7 (2.5)	0.409
Osteoarthritis	355 (55.6)	212 (59.9)	143 (50.4)	0.020
Chronic pulmonary diseases	18 (2.8)	6 (1.7)	12 (4.2)	0.093
eGFR < 60 mL/min/1.73 m^2^	224 (35.1)	148 (41.8)	76 (26.8)	< 0.001

Categorical variables are described as frequencies (n) and percentages (%). Age is presented as median and interquartile range (25–75th percentile). BMI is described using means and standard deviations.

Chi-square test or Fisher’s exact test was used to compare categorical variables. The Student’s t-test was used to compare two means of BMI. The Mann–Whitney test was used to compare two medians of age.

*eGFR* estimated glomerular filtration rate, *BMI* body mass index.

### Differences in main characteristics between the Fried frailty phenotype groups

To further understand the characteristics of frailty in older outpatients with T2DM, we compared the participants according to the frailty phenotype (Table 2). There was a trend for increasing age between the three groups with the median age of non-frail, pre-frail, and frail individuals being 68, 70, and 76 years, respectively (*P* < 0.001). Furthermore, there were significant differences between the three frailty phenotype groups in terms of sex, BMI, and productive work. Women (47.2%) and BMI < 20 kg/m^2^ (22.2%) were the most prevalent, whereas those with any form of job (1.7%) were the lowest in the frail group. Although there were no statistically significant differences among the three groups in marital status, level of education, living alone, polypharmacy, multimorbidity, and medical disorders, the rate of divorced/widowed was higher in the frail group than in the pre-frail and non-frail groups (31.1%, 23.0%, and 16.7%, respectively), and there were fewer highly educated older adults in the frail group than in the pre-frail and non-frail groups (52.8%, 60.2%, and 65.8%, respectively). Hypertension, osteoarthritis, and chronic kidney disease were the three most prevalent medical disorders, and were more frequently reported in the frail group.

**Table 2:** Characteristics of the participants according to the Fried frailty phenotype.

Characteristics	All (n = 638)	Nonfrail (n = 114)	Prefrail (n = 344)	Frail (n = 180)	*P*-value
Age, years	71.0 (66–77)	68.0 (64–74)	70.0 (65–76)	76.0 (69–80)	< 0.001
Age ≥ 75 years, n (%)	241 (37.8)	27 (23.7)	112 (32.6)	102 (56.7)	< 0.001
Men, n (%)	354 (55.5)	76 (66.7)	183 (53.2)	95 (52.8)	0.028
**Marital status, n (%)**					0.056
Single	7 (1.1)	2 (1.8)	4 (1.2)	1 (0.6)	
Married	477 (74.8)	93 (81.5)	261 (75.8)	123 (68.3)	
Divorced/Widowed	154 (24.1)	19 (16.7)	79 (23.0)	56 (31.1)	
Higher education, n (%)	377 (59.1)	75 (65.8)	207 (60.2)	95 (52.8)	0.072
Living alone, n (%)	72 (11.3)	18 (15.8)	36 (10.5)	18 (10.0)	0.269
Productive work, n (%)	74 (11.6)	32 (28.1)	39 (11.3)	3 (1.7)	< 0.001
BMI, kg/m^2^	22.9 ± 3.1	23.2 ± 2.6	23.3 ± 3.3	22.3 ± 3.1	0.004
**BMI groups, n (%)**					0.006
< 20	101 (15.8)	9 (7.9)	52 (15.1)	40 (22.2)	
20–24.9	379 (59.4)	78 (68.4)	193 (56.1)	108 (60.0)	
25–29.9	147 (23.1)	26 (22.8)	91 (26.5)	30 (16.7)	
30–34.9	11 (1.7)	1 (0.9)	8 (2.3)	2 (1.1)	
Polypharmacy, n (%)	367 (57.5)	63 (55.3)	191 (55.5)	113 (62.8)	0.239
Multimorbidity, n (%)	591 (92.6)	105 (92.1)	317 (92.2)	169 (93.9)	0.741
**Medical disorders, n (%)**					
Hypertension	566 (88.7)	100 (87.7)	301 (87.5)	165 (91.7)	0.316
Coronary artery disease	172 (27.0)	26 (22.8)	93 (27.0)	53 (29.4)	0.452
Heart failure	8 (1.3)	1 (0.9)	2 (0.6)	5 (2.8)	0.092
Stroke	21 (3.3)	5 (4.4)	6 (1.7)	10 (5.6)	0.052
Osteoarthritis	355 (55.6)	57 (50.0)	191 (55.5)	107 (59.4)	0.283
Chronic pulmonary diseases	18 (2.8)	1(0.9)	10 (2.9)	7 (3.9)	0.244
eGFR < 60 mL/min/1.73 m^2^	224 (35.1)	38 (33.3)	110 (32.0)	76 (42.2)	0.062

Categorical variables are described as frequencies (n) and percentages (%). Age is presented as median and interquartile range (25–75th percentile). BMI is described using means and standard deviations.

Chi-square test or Fisher’s exact test was used to compare categorical variables. The one-way ANOVA was used to compare three means of BMI. The Kruskal–Wallis test was used to compare three medians of age.

*eGFR* estimated glomerular filtration rate, *BMI* body mass index.

### Differences in Fried frailty phenotype components between men and women

In this study, frailty status varied according to sex. Frailty was more prevalent in women than in men (29.9% and 26.8%, respectively), but the difference was not statistically significant for the category of two states of the Fried frailty phenotype (*P* = 0.388) (Table 3). Of the five Fried frailty phenotype components, the proportion of participants with low grip strength was the highest (68.0%). This criterion was also the most prevalent among the men and women’s groups. Compared with the men’s group, the women’s group had higher rates of lower grip strength (75.4% vs. 62.1%, *P* = 0.001) and low walking speed (44.7% vs. 26.8%, *P* < 0.001), but a lower rate of less physical activity (31.3% vs. 43.8%, *P* = 0.002).

**Table 3:** Fried frailty phenotype and its components according to sex.

Characteristics	All (n = 638)	Men (n = 354)	Women (n = 284)	*P*-value
**Fried frailty phenotype (3 states), n (%)**				0.028
Non-frail	114 (17.9)	76 (21.5)	38 (13.4)	
Pre-frail	344 (53.9)	183 (51.7)	161 (56.7)	
Frail	180 (28.2)	95 (26.8)	85 (29.9)	
**Fried frailty phenotype (2 states), n (%)**				0.388
Non-frail^a^	458 (71.8)	259 (73.2)	199 (70.1)	
Frail	180 (28.2)	95 (26.8)	85 (29.9)	
**Components of Fried frailty phenotype, n (%)**				
Weight loss	117 (18.3)	64 (18.1)	53 (18.7)	0.931
Low grip strength	434 (68.0)	220 (62.1)	214 (75.4)	0.001
Exhaustion	142 (22.3)	70 (19.8)	72 (25.4)	0.112
Low walking speed	222 (34.8)	95 (26.8)	127 (44.7)	< 0.001
Low physical activity	244 (38.2)	155 (43.8)	89 (31.3)	0.002

Categorical variables are described as frequencies (n) and percentages (%).

Comparisons were conducted using the chi-square test.

^a^The non-frail and pre-frail groups were pooled together in one non-frail group.

### Associated factors of frailty in older outpatients with T2DM

Univariate and multivariate logistic regression analyses were performed to identify the potential factors associated with frailty (Table 4). In the adjusted model, two factors that increased the odds of frailty were older age and BMI < 20 mg/m^2^. In contrast, higher education and productive work were inversely associated with frailty. While all four factors were associated with frailty in women, only age and productive work were associated with frailty in men (Table 5).

**Table 4:** Factors associated with frailty in the logistic regression analysis (n = 638).

Variables	Univariate		Multivariate	
	OR (95% CI)	*P*-value	Adjusted OR (95% CI)	*P*-value
Age	1.09 (1.07–1.13)	< 0.001	1.08 (1.05–1.11)	< 0.001
Women	1.17 (0.82–1.65)	0.388		
Living alone	0.83 (0.47–1.46)	0.521		
Productive work	0.09 (0.03–0.30)	< 0.001	0.11 (0.03–0.36)	< 0.001
Higher education	0.70 (0.49–0.99)	0.042	0.64 (0.42–0.98)	0.041
**Marital status**				
Single	1 (reference)			
Married	2.10 (0.25–17.49)	0.498		
Divorced/Widowed	3.43 (0.40–29.21)	0.260		
**BMI groups**				
20–24.9	1 (reference)		1 (reference)	
< 20	1.65 (1.04–2.60)	0.033	1.96 (1.16–3.32)	0.012
25–29.9	0.64 (0.41–1.02)	0.060	0.70 (0.43–1.15)	0.157
30–34.9	0.56 (0.12–2.62)	0.460	0.64 (0.13–3.21)	0.586
Polypharmacy	1.36 (0.95–1.93)	0.093		
Multimorbidity	1.31 (0.65–2.64)	0.448		
Hypertension	1.56 (0.86–2.84)	0.142		
Coronary artery disease	1.19 (0.81–1.74)	0.375		
Osteoarthritis	1.24 (0.88–1.76)	0.226		
eGFR < 60 ml/min/1.73 m^2^	1.53 (1.07–2.18)	0.019		

Variables that had a *P*-value < 0.2 in the univariate regression were included in the multiple regression. Only variables that had a *P*-value < 0.05 in the multiple regression are shown.

*eGFR* estimated glomerular filtration rate, *BMI* body mass index, *CI* confidence interval, *OR* odds ratio.

**Table 5:** Factors associated with frailty in the logistic regression analysis according to sex.

Variables	Men (n = 354)		Women (n = 284)	
	Adjusted OR (95% CI)	*P*-value	Adjusted OR (95% CI)	*P*-value
Age	1.06 (1.03–1.10)	0.001	1.11 (1.06–1.16)	< 0.001
Productive work	0.08 (0.01–0.62)	0.015	0.19 (0.04–0.89)	0.035
Higher education			0.41 (0.21–0.83)	0.013
**BMI groups**				
20–24.9			1 (reference)	
< 20			2.43 (1.12–5.30)	0.025
25–29.9			0.42 (0.18–1.01)	0.053
30–34.9			0.61 (0.11–3.51)	0.578

Variables that had a *P*-value < 0.2 in the univariate regression were included in the multiple regression. Only variables that had a *P*-value < 0.05 in the multiple regression are shown.

*BMI* body mass index, *CI* confidence interval, *OR* odds ratio.

## Discussion

Frailty is an expression of physiological impairments and decreased functional reserve in multiple organ systems as a part of the ageing process, whereas T2DM is a pathological dysfunction characterised by hyperglycaemia and insulin resistance. These two clinical states can appear concurrently or consecutively in older adults^2^. They share several common pathophysiological mechanisms, such as metabolic impairment, increased oxidative stress, inflammatory dysregulation, and sarcopenia^27^. The presence of frailty in older patients with T2DM can increase the likelihood of adverse events and mortality^28,29^, whereas pathological metabolic changes in T2DM can increase the likelihood of frailty in older adults^27^. Nevertheless, sex differences in the patterns of frailty among older patients with T2DM are not fully understood. The results of this study provide three key observations. First, the prevalence of frailty in older outpatients with T2DM in Vietnam was 28.2%. There was no significant difference in prevalence of frailty between men and women. Second, there were differences in some characteristics between the men’s and women’s groups. Third, older age, productive work, higher education, and BMI < 20 kg/m^2^ were associated with frailty in the total study population and women, but only the first two factors were associated with frailty in men.

In a recent meta-analysis, the median community frailty prevalence using the frailty phenotype in individuals with diabetes was 13%, whereas the prevalence of frailty in outpatient populations varied widely due to heterogeneity in study settings, demographics, and especially in frailty assessment methods and differences in how frailty components were specified^2^. Our multicentre study is the first in Vietnam to find that the prevalence of frailty in older outpatients with T2DM was 28.2% when the Fried phenotype was used. Despite the differences in frailty assessment methods, our results are in accordance with the Action in Diabetes and Vascular Disease: Preterax and Diamicron Modified Release Controlled Evaluation (ADVANCE) trial using the frailty index and a recent study in Taiwan using the Tilburg frailty indicator showed that the prevalence of frailty in older outpatients with T2DM were 25.6% and 26.6%, respectively^30,31^. Since the benefits from intensive glucose-lowering and glycaemic goals were altered in patients with frailty^10,30^, the high overlap between the two states found in the previous studies and ours highlights the importance of identifying frailty in older patients with diabetes.

The physiological variability in the ageing process between men and women and sex differences in biological factors (e.g. inflammatory cytokines, abdominal adiposity, and cognitive impairment) and psychosocial factors (e.g. healthcare utilisation and self-reported behaviours) may interactively contribute to sex differences in frailty^32^. A meta-analysis showed that older women had higher frailty index scores than older men^12^. However, the female sex as an associated factor of frailty was found in some studies, but no significant association was found in other studies^33,34^. Among older adults with T2DM, a recent study revealed a higher rate of frailty in women than in men, but sex was not a significant factor associated with frailty^31^. Based on the positive association between age and frailty^33^, the greater prevalence of frailty in our women’s group may be related to a higher mean age in the women’s group than in the men’s group. Further research in a larger population and long-term follow-up is needed to elucidate the presence or absence of an association between the female sex and frailty.

Previous evidence from the Study of Osteoporotic Fractures showed that older women with diabetes had a greater decline in walking speed, but not in handgrip strength, than older women without diabetes^35^. This finding can be explained by the results from the Health, Aging, and Body Composition Study showing a decline in leg muscle strength and quality, but no differences in arm muscle strength and quality between older adults with and without T2DM^36^. However, interestingly, our study revealed that low grip strength was the most frequent component of the frailty phenotype in older adults with T2DM (68.0%), but not low walking speed. Our contradictory findings can be explained by variations within the Fried frailty criteria used, especially in handgrip strength protocols. Although handgrip strength is a reliable assessment of muscle weakness, the values of this method could be influenced by many factors, such as different dynamometer, posture and arm position, dominant and non-dominant hands, and the differences in normative reference values stratified by the BMI cut-off points between world regions^37,38^. Our study used the original Fried frailty criteria with grip strength stratified by the BMI cut-off points for developed regions^25^, but not for developing regions such as Vietnam. This may explain the high rate of weakness assessed by grip strength in our study population. While Tamura et al.^39^ set the cut-off points for weakness as handgrip strength < 26 kg in men and < 18 kg in women regardless of the BMI, they found 49.5% of older outpatients with cardiometabolic disease having low grip strength. It was also the most frequent component of the Fried phenotype.

Consistent with two previous studies including older adults with diabetes^39,40^, our study found significantly lower handgrip strength and slower walking speed in women than in men. These findings can be explained by several mechanisms. First, muscle mass and strength are determined by metabolic characteristics based on the regulation of sex hormones in nutrient sensing and the metabolism of organic compounds. In men, higher basal insulin levels promote more glycogen synthesis in muscle cells, and higher testosterone levels coupled with upregulated insulin-like growth factor signalling, results in greater muscle mass and strength^41^. A lower skeletal muscle mass than men, and oestrogen deficiency upon menopause negatively affects skeletal muscle protein turnover in women^42,43^. Second, in the older population, insulin resistance which can result in protein degradation, is associated with decreased quadriceps muscle strength and is a major risk factor for sarcopenia^44,45^. A recent study revealed that only older women with diabetes showed a higher prevalence of sarcopenia than those without diabetes, but these were not different in older men^40^. Third, women have a higher percentage of body fat than men, and there are sex differences in fat distribution. While men tend to have a central fat distribution with more abdominal visceral fat, women have a peripheral fat distribution with greater adipose tissue in the hips and thighs^46^. The data from the Framingham Heart Study revealed the impact of fat distribution on physical strength when intramuscular fat was associated with increased odds of low walking speed^47^. Taken together, the mechanisms may explain why men are faster and stronger than women, but other factors still contribute to the differences in physical performance between older men and women with T2DM (Table S1).

Frailty is a geriatric syndrome that is affected simultaneously by many sociodemographic, physical, biological, and psychological factors. Identifying these factors may help geriatricians recognise those with a high likelihood of frailty. Importantly, understanding the disparities in the factors associated with frailty between men and women, may be necessary to develop an individualised approach for frailty prevention and management^12^. Our study found that age, BMI, working status, and levels of education were factors associated with frailty. First, the findings are consistent with previous studies showing a positive association between frailty and increased chronological age in the overall older population, based on epidemiological evidence and biological mechanisms^33,48^. The putative mechanisms of increased susceptibility to frailty with ageing include loss of proteostasis, genomic instability, inflammation, epigenetic alterations, loss of stem cell regeneration, telomere shortening, deregulated nutrient sensing, and mitochondrial dysfunction^49^. Second, BMI is an important physical factor with a U-shaped association with frailty, in that BMI ≥ 25 kg/m^2^ and BMI < 20 kg/m^2^ were associated with a higher prevalence of frailty in older adults^50^. However, little is known about the relationship between BMI and frailty in the older population with diabetes. Although excess body weight is a major risk factor for T2DM, there was a significantly decreased risk of mortality in overweight patients as compared with normal weight patients, and the survival benefits of obesity were only detected in older patients^51^. Our study found that only underweight, defined as BMI < 20 kg/m^2^, was associated with frailty in older patients with T2DM. Importantly, this association was only present in the women’s group. This may be related to the fact that older men have a greater percentage of skeletal muscle mass than older women^52^, and BMI is inadequate to reflect older adults' strengths. Further studies are needed to understand the impact of BMI on frailty among older adults with T2DM.

The two protective factors of frailty found in our study were productive work and higher education. Previous studies have shown that older adults who continue to work or engage in any productive work beyond the retirement age are less likely to become frail. Prolonged work participation may offer a sense of independence and social connections for older individuals^53^. However, until now, the impact of the type and density of work on frailty remains elusive. In addition to productive work, many studies have revealed that higher education levels can place older adults at a lower risk of being frail^54,55^. Although education is not directly related to the pathophysiology of frailty, a better education may impact an individual’s lifestyle, such as increased awareness of healthy behaviours or increased ability to access social support and health services that may influence the progression of frailty^55^. Interestingly, higher education was a protective factor only in women, but not in men in the current study. This finding may require more insight into the sex-related preventive effects of higher education in the older population.

Our study has several limitations. First, the prevalence of frailty and burden of medical disorders in our study may not be generalisable to the general older adult population since the sample only included patients visiting geriatric clinics. Second, the study was performed at only three geriatric clinics in Vietnam. The results of our study may not be transferable to the general population or to other countries. Third, the impact of comorbidities and marital status on frailty was not fully evaluated because of the low rates of some diseases and the low rate of single participants in our study. Fourth, we were unable to evaluate the impact of specific antidiabetic agents and glycaemic goals on frailty because there were often switches of treatment regimens for diabetes and different individualised HbA1c goals in every patient. Fifth, due to the cross-sectional nature of the study design, we could not evaluate causal relationships between frailty and the associated factors. Further longitudinal studies are warranted to clarify these relationships.

## Conclusions

This is the first study to determine the prevalence of frailty in older patients with T2DM visiting geriatric clinics in Vietnam. Our findings add to the literature by demonstrating that older age and BMI < 20 kg/m^2^ were associated with increased odds of frailty, whereas higher education and productive work were associated with decreased odds of frailty. The sex differences in frailty in our geriatric outpatients with T2DM may suggest appropriate sex-related approaches to the management of frailty in these patients.

## Supplementary Information

Supplementary Information.

## Data Availability

The datasets used and/or analysed during the current study are available from the corresponding author on reasonable request.
