# Supplementary material for: Sex differences in frailty of geriatric outpatients with type 2 diabetes mellitus: a multicentre cross-sectional study
Source: Sci Rep. 2022 Sep 27;12:16122. doi: 10.1038/s41598-022-20678-7 (PMC9515181; doi:10.1038/s41598-022-20678-7)
Supplement: Supplementary file 1 — Supplementary Information. [file 41598_2022_20678_MOESM1_ESM.doc]

**Supplementary Information**

**Supplementary Table S1.** Vietnamese language version of Fried criteria

| **Thành tố** (Components) | **Cách đo đạc** (Measurement) |
| --- | --- |
| **Sụt cân không chủ ý**  (Unintentional weight loss) | Trong năm qua giảm không chủ ý >10 pound (>4.5kg) |
| **Yếu cơ**  (Weakness) | Dựa vào sức mạnh nắm tay được đo bằng dụng cụ Jamar 5030-J1  Hydraulic Hand Dynamometer (điểm cắt tùy theo giới và BMI) |
| **Mau mệt**  (Exhaustion) | Dựa vào 2 câu hỏi: “Trong tuần qua tôi làm mọi thứ đều  gắng sức” và “Trong tuần qua tôi không thể làm được gì” |
| **Chậm chạp**  (Slowness) | Thời gian đi 15 feet (4.57m) kéo dài hiệu chỉnh theo giới và tuổi |
| **Mức hoạt động thấp**  (Low physical activity) | Đối với nam tiêu hao ≤ 383 Kcal/tuần và nữ ≤ 270 Kcal/tuần  dựa vào bảng câu hỏi phiên bản rút gọn 18 hoạt động trong  Minnesota Leisure Time Activity |

The Vietnamese version was validated for using in two previous studies in Vietnam.

**References**

1 Vu, H. T. T. *et al.* Prevalence of frailty and its associated factors in older hospitalised patients in Vietnam. *BMC Geriatr* **17**, 216, doi:10.1186/s12877-017-0609-y (2017).

2 Nguyen, A. T. *et al.* Frailty Prevalence and Association with Health-Related Quality of Life Impairment among Rural Community-Dwelling Older Adults in Vietnam. *Int J Environ Res Public Health* **16**, doi:10.3390/ijerph16203869 (2019).
